# Hepatitis C Virus Infection: Looking for Interferon Free Regimens

**DOI:** 10.1155/2013/825375

**Published:** 2013-04-09

**Authors:** J. González-Moreno, A. Payeras-Cifre

**Affiliations:** Internal Medicine-Infectious Diseases Department, Hospital Son Llàtzer, Carretera de Manacor, Km 4, 07198 Palma de Mallorca, Spain

## Abstract

Recent developments of new drugs' combinations are changing the treatment paradigm in hepatitis C virus infection. Due to the side effect profile of pegylated interferons, interferon-sparing regimens have become the main target in chronic hepatitis C treatment research. Recent proofs of concept studies have suggested that cure of chronic hepatitis C can be achieved without interferon. The purpose of this paper is to provide an overview of the clinical results recently reported for the treatment of hepatitis C virus infection with interferon-free regimens, focusing on the most promising new compounds and combinations.

## 1. Introduction 

Hepatitis C virus (HCV) is a leading cause of liver failure, hepatocellular carcinoma and the most common reason for liver transplantation and infects about 3% of the population worldwide (approximately 170 million people chronically infected) [1]. The epidemic of injection drug use in the 1970s and 1980s and transmission via blood transfusions before 1992 are the main causes of disease expansion. The effect of longstanding infection and the aging process has been a significant increase in HCV-related cirrhosis and premature deaths over the past decade despite the decreasing incidence of new infections [2]. Recent development of new direct-acting antivirals (DAA) and preliminary results of trials using new treatments have opened up a new perspective in HCV therapy. When assembling optimal treatment combinations, factors that are important include the safety and tolerability profile of each agent, compatible pharmacokinetic profiles, and a low potential for unfavourable drug-drug interactions. It is know that interferon (IFN) has major common adverse effects and a bad security profile. That is why IFN-free regimens have become one of the priorities of researchers.

## 2. Natural History of Infection and Development of Actual Standard Therapy 

Currently, the primary causes of HCV transmission are intravenous drug use and, less frequently, unsafe medical or surgical procedures. The risk of vertical perinatal transmission is low (up to 5%) [3]. Although infrequent, some data suggest sex transmission in male who have sex with male. However, up to 44% of patients with acute HCV infection have no obvious risk factors [4]. 

Up to 75–85% of persons with acute HCV will develop chronic viral infection and between 5 and 25% will progress to cirrhosis over the following decades [5, 6]. The speed of histological deterioration is independent of viral genotype and viral load (VL), but is related to host factors such as male gender, obesity, age >40 at exposure, presence of concomitant liver disease unrelated to HCV as hepatitis B virus (HBV) infection, coinfection with human immunodeficiency virus (HIV) and life style aspects (daily intake of alcohol >50 g) [7, 8].

HCV has six different genotypes (GT) and more than 11 subgenotypes, in which genotype 1, with its subgenotypes 1a and 1b, is the most prevalent [10].

Initially the treatment for HCV was IFN monotherapy, administered daily. Soon it was found that the addition of ribavirin (RBV), an oral nucleoside analogue dosed twice a day, significantly increased viral responsiveness [11]. In the late 1990s, the advent of a pegylated form of IFN (pegIFN) allowed a weekly injection with sustained serum levels and a decrease in adverse effects. In the early 2000s, the efficacy of pegIFN alfa-2a and pegIFN alfa-2b when given with RBV was demonstrated, and this dual therapy (pegIFN-RBV) quickly became standard of care [12]. Although some clinical trials suggest the superiority of pegIFN alfa-2a over pegIFN alfa-2b, others found that both agents are equally effective, and no conclusive evidence supports the preferential use of either pegIFN [13, 14].

Cure in the setting of HCV pharmacotherapy is defined by sustained viral response (SVR), defined as the lack of HCV RNA in the serum 24 weeks after completion of antiviral therapy. Some predictors of SVR are well described and are summarized in Table 1. One of the most important pretreatment predictors of virologic response is the IL-28B polymorphism, described by Ge et al., in 2009 [15]. It is a single-nucleotide polymorphism, rs12979860 on chromosome 19, which represents the interferon lambda 28-B gene encoding IL-3.

Overall SVR rates achieved with pegIFN-RBV were clearly suboptimal at 40–50% for GTs 1 and 4. They were better for GTs 2 and 3 (more than 80%) [16]. Furthermore, this dual treatment has major common adverse effects, particularly hemolytic anemia with RBV and a wide spectrum of effects like neuropsychiatric symptoms or flulike fatigue and neutropenia with IFN, representing major impediments to adherence. 

The optimal duration of previous standard therapy with pegIFN-RBV depended on the viral genotype. Patients with genotypes 1, 4, 5, and 6 are generally treated for 48 weeks with pegIFN alfa-2a (180 mcg/weekly) or pegIFN alfa-2b (1–1.5 mcg/kg body weight/weekly) and weight-based daily dose of ribavirin (800–1200 mg). For genotypes 2, and 3 treatment could be completed within 24 weeks.

IFN and RVB still are the standard therapy for non-genotype 1 HCV infection, however recently this standard has changed for genotype 1 infection. 

## 3. Actual Standard Therapy for Genotype 1 HCV Infection 

An important step in HCV viral replication involves the nonstructural serine protease NS3-NS4A [17]. This protease induces expression of interferon *β* and leads to the expression of many interferon stimulated genes, thus producing an antiviral state in infected and surrounding cells [18]. NS3-4A also reduces the intrahepatic production of interferon *γ*, which might impair the hepatic inflammatory response and contribute to viral persistence [19]. Hence, inhibition of NS3-4A might block viral replication and potentially restore suppressed interferon pathways. 

In 2011, the US Food and Drug Administration (FDA) and European Medicines Agency approved the first two linear protease inhibitors, boceprevir and telaprevir. Triple therapy using these drugs in combination with IFN and RBV have become the actual standard of care for GT 1 HCV infection. 

Main characteristics of telaprevir and boceprevir are showed in Table 2. A brief summary of the main clinical trials using both drugs is described below. 

### 3.1. Telaprevir (TVR)

TVR was firstly described in clinical trials in 2009 [20, 21]. The first phase 3 study was published in 2011 [22]. In this study treatment naive GT 1 infected patients achieved SVR rates of 75% with triple therapy using TVR, as compared with a 44% SVR rate after 48 weeks of standard pegIFN-RBV. As in the ADVANCE trial [22] response guided therapy (RGT) was explored in the ILUMINATE trial [23]. In this study the overall SVR rate for all patients was 72%. For those with undetectable HCV RNA at treatment week 4 and week 12 or extended rapid virological response (eRVR), benefit was not found of extending therapy beyond an additional 4 weeks, as results were comparable (92% for 24 weeks treatment and 88% for 48 weeks treatment). SVR rates in non eRVR patients were only 64%, showing the importance of rapid virological response (RVR). In these studies a low proportions of patient with cirrhosis were included, so there are insufficient data for confident assessments of treatment regimens in advanced fibrosis. In the other hand other special populations showed interesting results in the ADVANCE trial [22]. For example, the SVR rate for patients with GT 1a was consistently lower than for 1b (71 versus 79%). Also black patients had lower SVR rates than non-black patients (62 versus 75%), although much higher than those receiving peg-IFNRBV alone (25%).

Treatment-experienced subjects were included in the phase 3 REALIZE study [24]. It showed that SVR rates were 83–88% for prior relapsers, 54–59% for previous partial responders, and 29–33% for null responders, as compared with 24, 15, and 5% for standard treatment, respectively. Treatment failures were associated with detectable resistant variants in 73% of cases. In contrast to the previous studies, the REALIZE trial included a higher portion of patients with cirrhosis (26%). Although cirrhotic patients had overall worse response, cirrhosis was not always associated with poor response (patients with cirrhosis who had previously relapsed achieved SVR 86% of the time).

### 3.2. Boceprevir (BOC)

In contrast with TVR trials, all studies of BOC include a 4-week lead-in phase of pegIFN alfa-2b and RBV alone followed by 24–44 weeks of triple therapy, allowing standard therapy to reach steady state in terms of anti-HCV effect before the direct acting antiviral. This lead-in phase was intended to minimise resistance and treatment failure by reducing viral replication before the addition of the third molecule [25].

First clinical trials were published in 2010 [26]. In phase 3 clinical trial SPRINT-2 [27], authors found SVR rates for standard therapy of 44% as compared with 67 and 68% for the arms using BOC. In this study, RGT was assessed and no significant differences were seen in SVR rates between the fixed-duration group (48 weeks of treatment) and the RGT group (28 weeks of treatment). It resulted in RGT recommendations in the product label for BOC.

The RESPOND-2 study [28] evaluated triple therapy with BOC in treatment-experienced patients for patients with some prior response to standard therapy (no previous null responders were enrolled). Overall SVR rates for the treatment arms receiving BOC were 59–66% (69–75% for prior relapsers; 40–52% for previous partial responders) as compared with 21% for standard therapy. Early response to triple therapy (undetectable RNA at week 8) was highly predictive of SVR (100%). 

## 4. Problems in Actual Treatment for Genotype 1 HCV Infection 

Main objections with actual triple therapy for GT 1 HCV infection, other than complex regimens and high cost, are summarized below. 

### 4.1. Side Effects and Drug Interactions

Because BOC and TVR must be combined with pegIFN and RBV, toxic effects and drug interactions associated with these protease inhibitors will be in addition to those of the previous standard of care. As a recent meta-analysis showed, TVR or BOC combined with pegIFN and RBV had favorable short-term data on SVR while resulting in more drug-related adverse effects [29]. Anemia is the most frequent side effect with either BOC or TVR [20–28]. How providers should treat anemia is unclear when using these new molecules either to use growth factors (off-label prescribing) or to reduce RBV dose. In addition, BOC and TVR are strong inhibitors of cytochrome P450 3A4 (CYP3A4), so important and commonly prescribed drugs can interact with them, like hormonal contraceptives, statins, dihydropirydine calcium channel blockers, and phosphodiesterase-5 inhibitors [25].

#### 4.1.1. Genotypes and Subgenotypes

Protease inhibitors are highly specific, and since the amino acid sequence of the NS3 protease domain differs significantly between HCV genotypes, their antiviral efficacy differs in different genotypes [30]. Due to the lack of findings from large clinical trials to support the use of TVR or BOC in patients with infections other than GT 1, both molecules should be prescribed only for GT 1 infection. Between subgenotypes, subgenotype 1a has lower rates of response than genotype 1b as showed in previous trials. The most probable reason for this disparity between subtypes is the higher genetic barrier to the development of protease inhibitor resistance that genotype 1b virus has [31].

#### 4.1.2. Drug Resistance

One of the most important lessons from the early studies with TVR and BOC is that used as monotherapy; these agents rapidly select for resistance variants, leading to virological failure. Naturally occurring dominant mutations resistant to the hepatitis C protease inhibitors are present even in previously untreated patients with GT 1 infection [31]. Because similar variants are detected in patients treated with either BOC or TVR, cross-resistance between these drugs is expected; thus, virological failure with triple therapy containing a protease inhibitor is a contraindication for a change from one drug to another.

## 5. Future Perspectives 

### 5.1. Role of Ribavirin

Some mechanism of action of RBV is well described [32]. It enhances Th1 CD4 responses increasing activity of cytotoxic T lymphocytes and secretion of antiviral cytokines such as interferon-*γ* and TNF-*α*. It also stops viral replication by inhibiting the formation of the guanosine nucleoside by inhibiting IMPDH (inosine monophosphatase dehydrogenase), inhibits the NS5B RNA-dependent RNA polymerase, and induces lethal mutagenesis by increasing errors in the translation of E2, NS5A, and NS5B. The role of ribavirin in IFN-based treatment is almost clear as it seems that RBV acts as a potentiator of host interferon-stimulated genes. It suggests that RBV may be uniquely capable of boosting host immune pathways in the cause of viral clearance [33]. 

Rates of SVR are lower in groups treated with pegIFN and a protease inhibitor than in people given pegIFN and RBV. For example, in the PROVE3 phase 2 trial of TVR for previously treated patients [34], the triple-therapy group had a rate of SVR of 53% compared with 24% in those given pegIFN alfa and TVR, half of those given pegIFN and RBV. In another study [20], viral breakthrough and relapse were also higher in the group of treatment without RBV: 24% and 48% versus 1–6% and 14–30% for the triple-therapy group, respectively.

Although the side effects of ribavirin are attenuated when taken without IFN, they still remain a problem. These include rash, cough, and haemolysis, with only a minor reduction in haemoglobin, unlike that seen in pegIFN-RBV regimens.

With the aim of improving RBV profile security an analogue have been proved in some clinical trials. Taribavirin is an RBV prodrug with a similar spectrum of antiviral activity but with better hepatocyte specificity and less accumulation in erythrocytes. It showed to reduce the anemia that is associated with RBV therapy without decreasing SVR rates [35]. Unfortunately, poorer results were noted in the larger ViSER2 study in which whilst taribavirin did cause less anemia, noninferiority was not achieved [36].

It also appears that RBV will continue to be required to suppress the emergence of viral resistance to DAAs in IFN-free regimens until more potent agents and oral combinations can be found. For example, in a study of Zeuzem et al. [37], the addition of RBV to the direct acting antivirals tegobuvir and GS-9256 substantially reduced the rate of resistance during treatment and also improved the median drop in HCV levels. Results from the 10 patients with HCV genotype 2 or 3 infection who received sofosbuvir alone (with lesser rate of SVR than in the group with RBV) in the study of Gane et al. [38] also suggest a role for ribavirin in the maintenance of an antiviral response. 

In the ZENITH study [39], both dual regimens (without RBV) were terminated after the arms met prespecified criteria for discontinuation because of a viral breakthrough rate >25% while no patient in either quad arm (using RBV) experienced viral breakthrough. This observation supports a role for RBV in DAA combinations, especially when low genetic barrier agents are combined. Of note, HCV subtype 1a was present in 11/12 patients who experienced viral breakthroughs. 

### 5.2. Interferon-*λ*

IFN analogues have been proposed in order to avoid IFN alfa side effects and increase SVR rates. Interferon-*λ*, the most promising IFN analogue developed, has greater specificity than IFN-2a for the targeting of hepatocellular cells, so there is expectation to significantly reduce unwanted side effects. On the basis of initial clinical trials, interferon-*λ* appears to be superior to standard interferon with respect to SVR for patients with a much-reduced side effect profile. Zeuzem et al. [40] presented interim results from an ongoing randomized trial in 2011 and demonstrated that for treatment-naive patients with HCV 1–4, there was an increased rate of RVR using (using doses of 180 *μ*g or 240 *μ*g/week) as compared with standard pegIFN alfa-2a. Furthermore, the incidence of flu-like symptoms, musculoskeletal symptoms, and cytopenias was significantly lesser with the use of interferon-*λ*. 

Some recent in vitro data suggest that has pan-genotypic activity against HCV and a nonoverlapping resistance profile to DAAs. It has been also observed that interferon-*λ* showed an additive to synergistic effect in vitro on HCV replication when combined with various classes of DAAs, including asunaprevir, daclatasvir, and BMS-791325 [41]. Some phase 2 and phase 3 clinical trials using IFN lambda in combination with different DAAs are currently ongoing. 

### 5.3. Interferon-Free Regimens

During viral infection, some of the most prominent cytokines produced are IFNs. The importance of IFNs goes beyond their antiviral activities and includes numerous immunoregulatory functions that affect both innate and adaptive immunities. IFN-induced clearance of HCV is both cytolytic (clearance of HCV-infected hepatocytes) and noncytolytic (intracytoplasmic destruction of HCV without cell injury) [42].

The challenges of IFN-based treatments have remained in place during the first year of the DAA era. These include all of the previously reported contraindications and adverse effects of IFN and RBV therapy. Effective IFN-free regimens would change these problems and are now a priority of investigators. 

As noted before, IFN and also RBV are required to block the emergence of DAA resistant viral strains [20, 43] as first generation protease inhibitors are drugs with a low genetic barrier. The main problem with new DAAs development is that new drugs may have cross-resistance with some of the most common mutations seen with TVR and BOC (R155K and D168A) [44]. They have shown cross-resistance to faldaprevir, simeprevir, asunaprevir, and ABT-450. This means that probably second-generation PIs should not be used in patients who have failed current triple therapies. Because R155K mutation requires only one nucleotide exchange for subtype 1a versus two nucleotide exchanges for subtype 1b [45], probably 1a subtype will continue to confer a lower barrier to resistance for second-generation PIs as previously seen for current triple therapy.

#### 5.3.1. First Steps

In October 2010, Gane et al. published results from the INFORM-1 (INterferon-Free regimen fOR the Management of HCV) trial [46]. It was a randomised, double-blind, placebo-controlled, dose-escalation trial. The study included 88 standard of care treatment-naive and treatment-experienced (null and nonnull responder) HCV GT 1 infected patients without cirrhosis, renal impairment, or viral coinfection (HBV or HIV). Patients were assigned to a study drug treatment regimen (*n* = 74 over seven treatment groups; 73 received at least one dose of study drug) or to placebo (*n* = 14, all of whom received at least one dose). They received up to 13 days oral combination treatment with mericitabine, a nucleoside polymerase inhibitor (500 mg or 1000 mg twice daily), and danoprevir, an NS3-4A protease inhibitor (100 mg or 200 mg every 8 h or 600 mg or 900 mg twice daily), or placebo. Dose escalation was started in HCV treatment-naive patients; standard-of-care treatment experienced patients, including previous null responders, were enrolled in higher-dose danoprevir cohorts. The primary outcome was change in HCV RNA concentration from baseline to day 14 in patients who received 13 days of a combination treatment. In the highest-dose cohorts, five of eight treatment-naive patients and two of eight null responders achieved HCV RNA concentrations below the limit of detection (<15 IU/mL) and seven of eight treatment-naive patients and four of eight null responders showed HCV RNA concentrations below the limit of quantification (43 IU/mL). The median change in HCV RNA concentration from baseline to day 14 ranged from −3.7 to −5.2 log 10 IU/mL in the cohorts that received 13 days of combination treatment. The combination of mericitabine and danoprevir was well tolerated with no-treatment-related serious or severe adverse events. This short-term phase 1 study showed for the first time that an interferon-free regimen could suppress viral replication. It was previously observed that the combination of mericitabine with DAAs that have a lower barrier to resistance, such as protease inhibitors, reduces drug resistance in vitro [47]. The INFORM-1 study confirms this benefit in vivo because the combination of mericitabine and danoprevir prevented resistance-associated virological breakthrough that has been reported with monotherapy with a protease inhibitor, including danoprevir.

Later, Zeuzem et al. [48] enrolled thirty-two HCV genotype-1-infected treatment-naive patients with chronic HCV GT 1 infection to be randomly assigned to groups that were given 400 mg or 600 mg BI 207127 (a polymerase inhibitor) 3 times daily plus 120 mg faldaprevir (a protease inhibitor) once daily and 1000 to 1200 mg/day RBV for 4 weeks. Thirty-one patients completed all 4 weeks of assigned combination therapy. In the group given BI 207127 400 mg 3 times daily, the rates of virologic response were 47%, 67%, and 73% at days 15, 22, and 29, respectively. A higher rate of response was observed in patients with GT 1b compared with GT 1a infections. In the group given BI 207127 600 mg 3 times daily, the rates of virologic response were 82%, 100%, and 100%, respectively, and did not differ among GTs. One patient in the group given 400 mg 3 times daily had virologic breakthrough at day 22. The most frequent adverse events were mild gastrointestinal disorders, rash, and photosensitivity. There were no severe or serious adverse events; no patients discontinued therapy prematurely.

The ZENITH study [39] was designed to compare VX-222, a nonnucleotide polymerase inhibitor administered 400 mg twice daily, and TVR alone versus VX-222 and TVR with RBV (triple) or with pegIFN (quadruple) for treatment-naive GT 1 patients. RGT in this study was defined as completion of treatment at week 12 for those with undetectable VL at weeks 2 and 8. At week 24 interim analysis, of those in the quadruple arms who achieved RGT targets, 82–93% of subjects had SVR after 12 week of treatment (SVR12).

#### 5.3.2. Achieving SVR without IFN

Lok et al. published a phase 2 study [49] using an oral-only trial for GT 1 HCV in patients who were prior null responders to standard therapy. This is the first group to demonstrate SVR data with patients receiving a regimen that is completely free of pegIFN. They randomnly assigned 21 patients to receive the NS5A replication complex inhibitor daclatasvir (60 mg once daily) and the NS3 protease inhibitor asunaprevir (600 mg twice daily) alone (group A, 11 patients) or in combination with pegIFN alfa-2a and RVB (group B, 10 patients) for 24 weeks. They excluded cirrhotic patients. The SVR rate at week 48 after treatment for the group A was low (27%), lower than group B (90%), but it created the expectation which is that new oral regimens will result in improved SVR in subsequent years, allowing even IFN-free regimens. In this study also was important the significance of subgenotypes. Although there were only 2 patients with HCV GT 1b infection in group A, the observation that both of the patients had an SVR after treatment with two direct-acting antiviral agents alone may reflect a higher resistance barrier for this combination of drugs in patients with HCV GT 1b infection than in patients with HCV GT 1a infection. All six patients (GT 1a) who presented viral breakthrough while receiving therapy had resistance mutations to both antiviral agents. The inclusion of pegIFN alfa-2a and RVB with daclatasvir and asunaprevir provided sufficient antiviral activity to suppress the emergence of resistance, an observation that is consistent with in vitro data showing a synergism of IFN and RBV with both direct-acting antiviral agents [50].

A similar finding of a high rate of SVR among patients with HCV GT 1b infection was shown in a pilot study of combination therapy with asunaprevir and daclatasvir in 10 Japanese patients who had HCV GT 1b infection and who had not had a response to previous therapy with pegIFN and RBV (<2 log 10 reduction in HCV RNA level by week 12) [51]. In this phase 2a study, patients were treated with daclatasvir in combination with asunaprevir for 24 weeks. The primary endpoint was the proportion of patients with undetectable HCV RNA at 12 weeks posttreatment. All enrolled patiens were subgenotype 1b. All 9 patients who completed treatment had an SVR. HCV RNA also remained undetectable posttreatment in the patient who discontinued after 2 weeks as he developed hepatotoxicity. There was no viral breakthrough during treatment or relapse of HCV RNA posttreatment.

Another Japanese study using asunaprevir in an IFN-free trial has been recently published [52]. In this trial, 43 chronic HCV GT 1b infected patients, 21 null responders and 22 intolerant to or medically ineligible for pegIFN-RBV therapy, were enrolled. They received dual oral treatment for 24 weeks with daclatasvir and asunaprevir. The primary efficacy end point was SVR12. Overall, 76.7% of patients achieved SVR12 and SVR24, including 90.5% of null responders and 63.6% of ineligible/intolerant patients. There were no virologic failures among null responders. Three (13.6%) ineligible/intolerant patients experienced viral breakthrough and four (18.2%) relapsed posttreatment. Diarrhea, nasopharyngitis, headache, and ALT/AST increases, generally mild, were the most common adverse events; three discontinuations before week 24 were due to adverse events that included hyperbilirubinemia and transaminase elevations (two patients).

The antiviral activity of tegobuvir (a nonnucleoside NS5B polymerase inhibitor) and GS-9256 (an NS3 serine protease inhibitor) as oral combination therapy, or together with RVB or pegIFN alpha-2a and RBV, was assessed in a phase 2, randomized, open-label trial [37]. Treatment-naive patients with GT 1 HCV were assigned 28 days of tegobuvir 40 mg twice daily and GS-9256 75 mg twice daily (*n* = 16), tegobuvir and GS-9256 plus RBV 1,000–1,200 mg daily (*n* = 15), or tegobuvir and GS-9256 plus pegIFN alpha-2a (180 *μ*g once weekly)/RBV (*n* = 15). After 28 days, when RVR was evaluated, all patients received pegIFN-RBV. RVR was observed in 7% (1/15) of patients receiving tegobuvir/GS-9256, 38% (5/13) receiving tegobuvir/GS-9256/RBV, and 100% (14/14) receiving tegobuvir/9256/PEG-IFN/RBV. Authors concluded that in genotype 1 HCV, adding RBV or RBV with pegIFN provides additive antiviral activity to combination therapy with tegobuvir and GS-9256.

In 2011, Lawitz et al. showed that monotherapy with sofosbuvir (a polymerase inhibitor) at a dose of 400 mg for 7 days resulted in a profound reduction in the level of HCV RNA in patients with HCV GT 1 infection [53].

Gane et al. published recently the results from the ELECTRON trial [38], a phase 2a study designed to test the safety and efficacy of sofosbuvir and RBV in various IFN-sparing and IFN-free regimens for the treatment of patients with HCV GT 1, 2, or 3 infection. They did not included patients with cirrosis, HBVm or HIV. A total of 40 previously untreated patients with HCV GT 2 or 3 infection were randomly assigned to four groups; all four groups received sofosbuvir (at a dose of 400 mg once daily) plus RBV for 12 weeks. Three of these groups also received pegIFN alfa-2a for 4, 8, or 12 weeks. Two additional groups of previously untreated patients with HCV GT 2 or 3 infection received sofosbuvir monotherapy for 12 weeks or sofosbuvir plus pegIFN alfa-2a and RBV for 8 weeks. Two groups of patients with HCV GT 1 infection received sofosbuvir and RBV for 12 weeks: 10 patients with no response to prior treatment and 25 with no previous treatment. They found that viral kinetics during treatment were nearly identical in all treatment groups and that viral suppression was rapid in all patients, regardless of GT, status with respect to previous treatment, baseline VL, race or ethnic group, IL28B status, and presence or absence of IFN in the regimen. Of the 40 patients, all 10 who received sofosbuvir plus RBV without IFN and all 30 who received sofosbuvir plus RBV for 12 weeks and IFN for 4, 8, or 12 weeks had an SVR at 24 weeks. The presence or absence of pegIFN alfa-2a appeared to have no effect on viral kinetics or rate of SVR. For the other patients with HCV GT 2 or 3 infection, all 10 who received sofosbuvir plus pegIFN alfa-2a and RBV for 8 weeks had an SVR at 24 weeks, as did 6 of 10 who received sofosbuvir monotherapy. Among patients with HCV GT 1 infection, 21 of 25 previously untreated patients (84%) and 1 of 10 with no response to previous therapy (10%) had an SVR at 24 weeks. In the last group, most of the patients were GT 1a and had unfavourable IL28B allele types, and all of these patients relapsed when the 12-week oral regimen was completed. The most common adverse events were headache, fatigue, insomnia, nausea, rash, and anemia. The IFN-free arm demonstrated the lessening impact on haemoglobin when just RBV plus sofosbuvir was given. Furthermore, there was no effect on absolute neutrophil count when pegIFN alfa-2a was not included in the regimen. No patients discontinued sofosbuvir or RBV in any group.

Preliminary results have also been reported from a phase 2a study evaluating sofosbuvir and daclatasvir for 24 weeks with or without RBV in previously untreated patients with HCV GT 1, 2, or 3 infection [54]. All 44 patients with HCV GT 1 infection had an SVR at 4 weeks after treatment. Among patients with HCV GT 2 or 3 infection, rates of SVR at 4 weeks ranged from 88 to 100%.

Although the high SVR rates were showen using sofosbuvir in the previous studies for GT 1 treatment-naive patients, a press release of the QUANTUM study 24-week data in this population showed an SVR rate of only 59% [55]. So, it is likely that this regimen will not be adequate for genotype 1 patients.

Finally, Poordad et al. published recently a phase 2a study [56] in which the safety and efficacy of the combination of ABT-450/r and ABT-333 with RBV in previously untreated patients with HCV GT 1 infection and in patients with a null or partial response to previous treatment with pegIFN-RBV was assessed. They excluded patients with cirrhosis, renal impairment and coinfection with HBV or HIV. In total, 50 patients were included and all of them received ABT-333 (400 mg twice daily) and RBV (1000 to 1200 mg per day) and one of two daily doses of ABT-450/r. Patients were divided in to 3 groups. Groups 1 and 2 included previously untreated patients; group 1 received 250 mg of ABT-450 and 100 mg of ritonavir and group 2 received 150 mg and 100 mg, respectively. Group 3, which included patients who had had a null or partial response to previous therapy with pegIFN-RBV, received daily doses of 150 mg of ABT-450 and 100 mg of ritonavir. The primary endpoint was the percentage of patients with eRVR. A total of 17 of the 19 patients in group 1 (89%) and 11 of the 14 in group 2 (79%) had an eRVR; an SVR 12 weeks after the end of treatment was achieved in 95% and 93% of the patients, respectively. In groups 1, and 2 there were no virologic failures during treatment or during 48 weeks of followup for those who completed treatment. As expected, results were significantly worse in group 3. In this group, 10 of 17 patients (59%) had an eRVR, and 8 (47%) had an SVR 12 weeks after therapy; 6 patients had virologic breakthrough, and 3 had a relapse. Resistant variants in NS3 and NS5B were detected in eight patients who had virologic failure; one patient with a relapse had no variants at any signature position. No deaths or serious adverse events occurred during the study. Overall, the most frequent events were fatigue, nausea, headache, dizziness, insomnia, pruritus, rash, and vomiting. In contrast to other trials of IFN-free protease-inhibitor-containing combination therapy in previously untreated patients, where reduced activity against HCV GT 1a as compared with HCV GT 1b was found, no virologic failures occurred among the 31 previously untreated patients who completed treatment, including 26 patients with HCV GT 1a infection in this study. Regarding IL28B genotype in this study, in contrast to previous trials, more than half of the previously untreated patients who completed the study treatment had a CT or TT IL28B genotype, and all had an SVR.

Previous noted results strongly suggest that in the near future IFN-free regimens for HCV infection will become the standard treatment. With that aim there are currently some clinical trials ongoing, investigating new molecules combinations in IFN-free regimens (Table 3). 

### 5.4. Special Populations

#### 5.4.1. HIV Coinfection

Since the development and widespread application of effective medications for the treatment of HIV disease, coinfection with HCV has become an important cause of morbidity and mortality in HIV infected patients. People with HIV coinfection are more likely to progress to cirrhosis and suffer the complications of end-stage liver disease, hepatocellular carcinoma and death [57].

Eradication of HCV with therapy is associated with a regression of liver fibrosis and improved survival in HIV/HCV coinfected patients [58, 59].

The antiviral effects of IFN vary among coinfected patients, based on effects of HIV infection and/or disease, and many coinfected patients are not eligible for IFN based therapy due to comorbid conditions, particularly psychiatric diseases. The bone-marrow-suppressive effects of IFN are exacerbated in many HIV-infected patients, further complicating the treatment. The development of combination regimens of DAAs that are safe for coinfected patients is therefore of high priority.

Drug interactions between DAA and antiretroviral agents, selection of HCV drug resistance, poor drug adherence, and high cost are among the most important challenges that may arise when using DAA in coinfected patients.

Currently, poor clinical data about the use of DAAs in HIV/HCV-coinfected patients is available. 

Promising interim results of triple therapy using TVR are available from a study of coinfected patients with GT 1 [60]. Twelve weeks after treatment was completed, 74% of previously untreated patients with coinfection had undetectable hepatitis C RNA when treated with a TVR-based regimen, compared with 45% of patients treated with pegIFN plus RBV and placebo. Tolerability was comparable to that of TVR treatment in patients with hepatitis C monoinfection. The mean CD4 in this population was above 550 cells/mm^3^, and there were no drops in the percentage of these cells during therapy nor HIV-RNA rebounds in patients on antiretroviral therapy.

A trial testing BOC in HIV/HCV-coinfected patients included 98 patients that had reached 12 weeks posttreatment [61]. The SVR12 rate was 60.7% in patients treated with BOC plus pegIFN-RBV compared with 26.5% in patients treated with pegIFN-RBV alone. The mean CD4 count was above 600 cells/mm^3^, as all patients were on antiretroviral therapy (84% on HIV protease inhibitors). There were no drops in the percentage of CD4+ T-cells neither HIV-RNA rebounds. Overall, 14% of patients had to discontinue BOC due to serious adverse events, mainly anemia.

As some experts opine [62], available clinical data may support the use of BOC or TVR in coinfected patients with high CD4 T-cell counts who are not taking ART or those on select ART regimens for which sufficient safety data have been provided. At this moment, the off-label use of TVR may be considered in theory, in patients using atazanavir boosted with ritonavir, raltegravir or efavirenz (using higher TVR dose) based therapies in combination with tenofovir and emtricitabine. On the other hand, it is evident that BOC should not be combined with nonnucleoside reverse transcriptase inhibitors or ritonavir-boosted HIV protease inhibitors.

There are phase 3 clinical trials ongoing using faldaprevir, asunaprevir with daclatasvir, and simeprevir in combination with pegIFN-RBV in coinfected patients. To date there are no clinical trials using IFN sparing regimens in HIV/VHC-coinfected patients. 

#### 5.4.2. Nongenotype 1 Patients

Worldwide, 50–70 million subjects are infected with an HCV GT 2, 3, 4, 5, or 6 [63]. In these patients, the combination of pegINF and RBV remains the currently approved standard-of-care treatment and it is results are not optimal especially in GT 4 patients.

In 2011, Foster et al. observed reduced levels of HCV RNA in 9 GT 2 infected patients under treatment with TVR in monotherapy or with pegIFN-RBV [64]. TVR monotherapy or in combination in triple therapy had no activity in 8 genotype 3 patients studied.

In a phase 2a study conducted in 24 genotype 4 patients [65], triple therapy with TVR for 2 weeks induced a greater reduction in the HCV RNA level compared with the standard treatment, however, it did not lead to a greater SVR (62% in both groups).

Preclinical studies with simeprevir showed inhibition of HCV replication across all GT with an IC50 value below 13 nM except for GT-3a protease [66]. However, in a phase 2a trial it showed a weak activity in GT 2 infected patients [67].

Danoprevir has equipotent activity against HCV GT 1, 4, and 6 in vitro [68]. In the DAUPHINE trial [69], all 33 enrolled GT 4 patients achieved SVR12 with different doses of danoprevir boosted with ritonavir (DNVr) for 24 weeks, independently of the DNVr dose and the treatment. 

As cited before sofosbuvir showed in the ELECTRON tiral [38] excellent SVR rates for GT 2 and 3, specially in combination with RBV. 

In summary early results of clinical trials suggest that oral pegINF free treatments can lead to definitive clearance of the virus in most naive and treatment-experienced GT 2 and 3 HCV infected patients. Also triple therapy with sofosbuvir in combination with pegIFN-RBV have shown promising results for GT 4 HCV infection. Clinical results of the use of DAAs for GT 5 and GT 6 HCV infection are limited.

#### 5.4.3. Other Populations

Limited data is available for some other special populations. For example, triple therapy regimen studies were not performed on subjects with Child-Pugh score >7, and even if there are no data to suggest that TVR or BOC require dose adjustment in advanced cirrhosis, use of this regimen cannot be recommended while pegIFN is a component of the regimen. 

As noted previously, although cirrhotic patients had overall worse response in the REALIZE study [24], cirrhosis was not always associated with poor response. No decompensated cirrhotic patients were neither included in IFN free regimen studies. Trials that are large and include adequate numbers of patients with advanced fibrosis will be necessary to prove a drug regimen is competitive. As for cirrhotics, there are insufficient data on new HCV therapy regimens for postliver transplant, renally impaired, black, young (<18), and old (>65) patients. 

## 6. Conclusions 

Recent approval of two DAA drug for the treatment of HCV infection has opened a new perspective in HCV therapy. As a result of the side effects of IFN, there is ongoing search for effective IFN-free antiviral regimens. Promising data have been presented in proof-of-concept studies, confirming that SVR without using IFN may be feasible. Ideal drug regimen should work for all GTs and must prevent the emergence of drug resistant viral strains, have a high degree of safety and efficacy, an easy treatment algorithm, and short treatment duration. More studies are needed to support IFN-free regimens, particularly in some difficult-to-treat populations. 

## Figures and Tables

**Table 1:** Some classical of predictors of sustained viral response (SVR).

	Positive predictors for SVR
HCV-related factors	Genotypes 2 and 3
	Subgenotypes 1a and 2a
	Low pretreatment VL
	RVR, eRVR, EVR

Host-related factors	Young age
	Absence of advanced liver fibrosis
	Caucasians
	IL28B CC genotype

Treatment-related factors	No RBV dose reduction (dose > 10.6 mg/kg per day)
	Good adherence

eRVR: extended rapid virological response (undetectable HCV RNA at treatment week 4 and week 12); EVR: early virological response (HCV RNA detectable at week 4 but undetectable at week 12, maintained up to the end of treatment); HCV: hepatitis C virus; IL28B: interleukin 28B; RBV: ribavirin; RVR: rapid virological response (absence of measurable HCV RNA at week 4 of treatment); VL: viral load.

**Table 2:** FDA approved directly acting antiviral drugs—telaprevir (Incivek) and boceprevir (Victrelis). Modified from Assis and Lin [9].

	Telaprevir	Boceprevir
Formulation	375 mg oral capsule	200 mg oral capsule
Dosing	750 mg/7–9 h with a fatty meal*	800 mg every 7–9 h with food*
Regimen	12 weeks of triple therapy followed by Peg-IFN/ribavirin alone for 12 or 36 weeks**	Start after 4-week lead-in of Peg-IFN and ribavirin for 24 or 44 weeks**
Discontinuation	If VL >1,000 IU/mL at week 4 or 12, and VL at week 24	If VL >100 IU/mL at week 8, 12, and VL at week 24
Expected SVR	Naive: 69–75% Relapser: 84–88% Partial responder: 56–61% Null responder: 31–33%	Naive: 63–66% Relapser: 69–75% Partial responder: 40–52% Null responder: not studied
RGT	If negative VL at weeks 4 and 12, treat with Peg-IFN/ribavirin for 12 more weeks If not, treat for 36 more weeks**	Treatment-naive: if negative VL at week 8 and 24, complete treatment at week 28 Previously treated: if negative VL at week 8 and 24, complete treatment at week 36**
Barrier to resistance	Low (V3M6, R155K)	Low (V3M6, R155K)
Common adverse effects	Anemia (37%) Rash (56%; severe in 4–7%) Anal itching/burning (29%)	Anemia (49%) Dysgeusia (43%)
Metabolism	Hepatic (CYP450)***	Hepatic (CYP450 and aldoketoreductase)***
Interactions	Strong inhibitor of CYP3A	Strong inhibitor of CYP3A4/5 and is partly metabolized by CYP3A4/5
Renal/hepatic adjustment	Renal: none Hepatic: do not use if Child-Pugh score >7	Renal: none Hepatic: do not use if Child-Pugh score >7
FDA-labeled indications	Chronic HCV genotype 1a or 1b in combination with Peg-IFN and ribavirin, in adults with compensated liver disease (Child-Pugh <7)	Chronic HCV genotype 1a or 1b, in combination with Peg-IFN and ribavirin, in adults with compensated liver disease, including cirrhosis, who are previously untreated or who failed previous Peg-IFN/ribavirin treatment
Contraindications	Pregnancy (due to ribavirin) Coadministration with drugs highly dependent on CYP3A for clearance	Pregnancy (due to ribavirin) Coadministration with drugs highly dependent on CYP3A4/5 for clearance
Special populations	Not approved in decompensated cirrhosis, HIV-HCV or HBV-HCV coinfection, pediatrics, and posttransplant	Not approved in decompensated cirrhosis, HIV-HCV or HBV-HCV coinfection, pediatrics, and posttransplant

FDA: US Food and Drug Administration; HBV: hepatitis B virus; HCV: hepatitis C virus; IFN: interferon; R155K: lysine to arginine substitution at position 155; RGT: response guided treatment; SVR: sustained virologic response; VL: viral load; V36M: valine to methionine substitution at position 36. *No dose reduction is allowed because of risk of engender drug resistance. **48 weeks of treatment always for patients with cirrhosis and previous null responders. In telaprevir treatment also for partial responders. In boceprevir also for previously untreated patients who respond poorly to interferon in the lead-in period (<1 log 10 decline in hepatitis C RNA from baseline). ***Special care using first-generation protease inhibitors which are hormonal contraceptives, statins, dihydropyridine calcium channel blockers, and phosphodiesterase-5 inhibitors.

**Table 3:** Clinical trials ongoing using IFN-free regimens.

Drugs	DAA	Trial	Genotypes	Patients	Sponsor
Faldaprevir + BI207127 + Ribavirin	PI NS3/4A + NNPI NS5B	Phase 3	1	Treatment-naive	Boehringer Ingelheim
Simeprevir + TMC647055 + Ritonavir + Ribavirin	PI NS3/4A + Ritonavir potentiated NNPI NS5B	Phase 2a	1	Treatment-naive and previous relapsers	Janssen R & D
Simeprevir + Sofosbuvir + Ribavirin	PI NS3/4A + NPI NS5B	Phase 2a	1	Null responders	Janssen R & D
Asunaprevir + Daclatasvir	PI NS3 + NS5A inhibitor	Phase 3	1b	Treatment-naive	Bristol-Myers Squibb
ABT450 + Ritonavir + ABT267	Ritonavir potentiated PI NS3 + NS5A inhibitor	Phase 2	1b, 2	Treatment-experienced	AbbVie (prior sponsor, Abbott)
ABT450 + Ritonavir + ABT267 + ABT333	Ritonavir potentiated PI NS3 + NS5A inhibitor + NNPI NS5B	Phase 2	1	Treatment-experienced	Abbott
Danoprevir + Ritonavir + Mericitabine + Ribavirin	Ritonavir potentiated PI NS3/4A + NPI NS5B	Phase 2	1	Treatment-experienced	Hoffmann-La Roche
Sofosbuvir + GS5885 + Ribavirin	NPI NS5B + NS5A inhibitor	Phase 3	1	Treatment-experienced	Gilead Sciences
Sofosbuvir + Ribavirin	NPI NS5B	Phase 3	2, 3	Treatment-naive and Treatment-experienced	Gilead Sciences
Setrobuvir + Danoprevir + Ritonavir + Mericitabine	NNPI NS5B + Ritonavir potentiated PI NS3/4A + NPI NS5B	Phase 2	1	Treatment-naive and null responders	Hoffmann-La Roche
Asunaprevir + Daclatasvir + BMS791325	PI NS3 + NS5A inhibitor + NNPI NS5B	Phase 2	1	Treatment-naive	Bristol-Myers Squibb
Daclatasvir + Sofosbuvir + Ribavirin	NS5A inhibitor + NPI NS5B	Phase 2	1, 2, 3	Treatment-naive	Bristol-Myers Squibb
GS5885 + GS9451 + Tegobuvir + Ribavirin	NS5A inhibitor + PI NS3/4A + NNPI NS5B	Phase 2	1	Interferon ineligible or intolerant subjects	Gilead Sciences

DAA: direct acting antiviral; NNPI: nonnucleoside polymerase inhibitor; NPI: nucleoside polymerase inhibitor; PI: protease inhibitor.
